# Evaluation of the Models for Forecasting Dengue in Brazil from 2000 to 2017: An Ecological Time-Series Study

**DOI:** 10.3390/insects11110794

**Published:** 2020-11-12

**Authors:** Marcos Venícius Malveira de Lima, Gabriel Zorello Laporta

**Affiliations:** 1 Doctoral Program in Health Sciences at Centro Universitário Saúde ABC (FMABC), Fundação do ABC, Santo André, SP 09060-870, Brazil; marcos.malveira@ac.gov.br; 2 Postgraduate Sector, Research and Innovation, Centro Universitário Saúde ABC (FMABC), Fundação do ABC, Santo André, SP 09060-870, Brazil

**Keywords:** dengue, decision support techniques, epidemiological monitoring, forecasting, time-series studies

## Abstract

**Simple Summary:**

Dengue is an infectious disease that affects thousand millions of people worldwide every year. Here we applied statistical modeling for forecasting future epidemics in Brazil. Future predictions were possible in some Brazilian states and with particular models. We strongly recommend the use of the analysis protocol developed here on a routine basis in state health control services to predict dengue epidemics in Brazil.

**Abstract:**

We aimed to evaluate the accuracy of deterministic and stochastic statistical models by means of a protocol developed in a free programming environment for monthly time-series analysis of the incidence of confirmed dengue cases in the states and federal district of Brazil from January 2000 to December 2017. This was an ecological time-series study conducted to evaluate and validate the accuracy of 10 statistical models for predicting the new cases of dengue. Official data on the monthly cases of dengue from January 2000 to December 2016 were used to train the statistical models, while those for the period January–December 2017 were used to test the predictive capacity of the models by considering three forecasting horizons (12, 6, and 3 months). Deterministic models proved to be reliable for predicting dengue in a 12-month forecasting horizon, while stochastic models were reliable for predicting the disease in a 3-month forecasting horizon. We were able to reliably employ models for predicting dengue in the states and federal district of Brazil. Hence, we strongly recommend incorporating these models in state health services for predicting dengue and for decision-making with regard to the advanced planning of interventions before the emergence of epidemics.

## 1. Introduction

Dengue is an infectious disease with a wide range of symptoms [1]. The illness is classically characterized by high fever, headache, vomiting, myalgia, arthralgia, and rash. Dengue shares its symptoms with many other infectious diseases that are common in the tropical environment [2]. The disease can become severe in a small proportion of patients in whom vascular extravasation can be life-threatening [1]. In most cases of dengue fever (18–60%), the patients are asymptomatic [3]. However, such asymptomatic carriers may likely help in maintaining disease circulation and therefore pose a considerable challenge in disease surveillance and control [4,5]. Despite the mild manifestation of the disease in some individuals, the volume of cases under hospital care, particularly outpatient consultations, is the main factor responsible for the burden imposed by dengue on society. Absence from work and chronic problems such as depression and fatigue also add to the societal burden [6,7].

The dengue virus (DENV) is a single-stranded RNA virus and the member of the *Flavivirus* genus with the highest global prevalence. The virus is transmitted by *Aedes* (*Stegomyia*) Theobald mosquitoes, which generally occupy human-made habitats. This is particularly true for the main vector *Aedes* (*Stegomyia*) *aegypti* (Linnaeus), a species well-adapted to the current urban landscape [8]. Seasonality in DENV transmission within a year is affected by climatological factors. Higher rainfall indices from October to March every year contribute to the increase in the number of available breeding sites for this mosquito [9]. The rainy season increases the risk of cases of dengue in most of Brazilian cities [10]. Even though dengue is a typically seasonal disease, cases of the disease can be reported both in rainy and dry periods, as the reduction in the adult vector density in the colder and drier months is not enough to cease DENV transmission [10]. Adding to this difficulty in establishing a key seasonal pattern, the transmission of the disease throughout the year benefits from the complex dynamics of viral infections, including the availability of susceptible humans and the introduction of different DENV serotypes in the same area over the years [11]. For instance, DENV is classified into four genetically distinct strains (serotypes I–IV), and infection with one of them causes specific immunological responses in the human host and partial cross-immunity to the other serotypes [2]. Altogether, the broader pattern of dengue epidemic and interepidemic cycles is complex and therefore poses a challenge to the disease surveillance and control.

In tropical regions, particularly in Latin America, the DENV has become the most important human pathogen. Eggs of *Ae*. *aegypti* were periodically spread into the Americas by ships sailing the Atlantic Ocean continuously from the Old World. This contributed to the emergence of urban Yellow Fever in Brazil during the first half of the twentieth century. Considering that these urban epidemics had commonly been threatening people in cities, such as Rio de Janeiro, the Brazilian federal government launched eradication campaigns for eliminating this mosquito from urban scenarios. *Aedes aegypti* was officially declared eradicated in the country in the 1940s; however, the mosquito has been re-colonizing the entire country from the 1970s on [12]. Along with the continuous introductions of DENV serotypes in the country, the magnitude and geographical reach of dengue epidemics have been increasing over the years [13]. The last major dengue epidemics with 3.2 million cases reported in Brazil occurred in 2015–2016 [14]. Today all four serotypes (DENV-I–IV) have been circulating throughout the country, and more than 90% of the Brazilian municipalities are potentially infested by *Ae*. *aegypti* [15]. In other words, the “dengue problem” in Brazil is absolutely unsolvable with the current available tools. We further believe this is a problem common to all Latin American countries.

Predictive modeling of the incidence of confirmed dengue cases is an important tool for health surveillance as well as for planning control measures [16,17]. Here, we present a study to evaluate the accuracy of time-series statistical models in predicting symptomatic dengue in Brazilian states. The monthly time series of new cases can be adjusted in statistical functions with the application of computational algorithms [18,19,20]. The time series can be broken down into three basic components: a seasonal component that represents the cyclical pattern of the disease over time; a linear component that refers to the tendency of the disease to decrease or increase in a linear manner over time; and a stochastic component that refers to the intervening factors affecting the time series without a specific temporal pattern.

Time-series statistical models can be used to predict future cases. The autoregressive model with integrated moving average (ARIMA) is a pioneering method for describing and predicting the time series [21]. The exponential smoothing model (ETS) serves as an alternative to ARIMA [22]. Exponential smoothing models for adjusting complex seasonal patterns with trigonometric Box-Cox transformations and autoregressive moving average errors (TBATS and BATS) are considered to be more efficient than ARIMA [23]. An alternative model is the seasonal trend decomposition using Loess (STLM), which breaks down the seasonal component into subcomponents [24].

The models described above are deterministic and have the statistical structure to break down and adjust the seasonal and linear components of the time series; however, they cannot estimate the stochastic component. Computational approaches to machine learning were therefore proposed to quantify the effect of this component. The structural model (StructTS), neural network model (NNETAR), extreme learning machine and multilayer perceptron models (ELM, MLP) are all examples of stochastic models [21,25,26]. The models to be evaluated can be compared with the null model defined by the value of the last observation [27].

In this study, deterministic statistical models (those with superior performance in stable temporal patterns) and stochastic models (those with superior performance in chaotic temporal patterns) were used. The main assumption for model selection was having all selected models in a single, open-software analytic environment in order to help in the implementation of these tools for the state health secretaries in Brazil (Supplementary Material—Text S1). This study was based on the need to elaborate a routine in an open source programming environment, which can be adopted by the Brazilian state surveillance sector to predict future dengue epidemics. It is hoped that this endeavor will help in the preparation and planning of control measures in a timely and effective manner. This study aimed to evaluate the accuracy of different predictive methods in the time-series analysis of the incidence of confirmed symptomatic dengue cases in the 26 states and federal district in Brazil from 2000 to 2017. The evaluation focused on comparing and judging the merits of the available statistical models [21,22,23,24,25,26,27] for predicting the incidence of future cases in each Brazilian state and federal district. The real-time series of 2017 was used as the reference standard for calculating the predictive accuracies of the methods and comparing them.

## 2. Materials and Methods

### 2.1. Design

A statistical and computational approach was applied for evaluating and validating the accuracy of 10 statistical models (ARIMA, ETS, TBATS, BATS, STLM, StructTS, NNETAR, ELM, MLP, and null model) to predict the time series of new dengue cases. Official data on the monthly dengue cases from January 2000 to December 2016 were used to train the statistical methods, while those for the period January–December 2017 were utilized to test the predictive capacity of each model by considering three forecasting horizons (12, 6, and 3 months). Detailed information and rationale for each of the selected models in this study are provided in Supplementary Material—Text S1.

### 2.2. Study Sites

**Figure 1:**
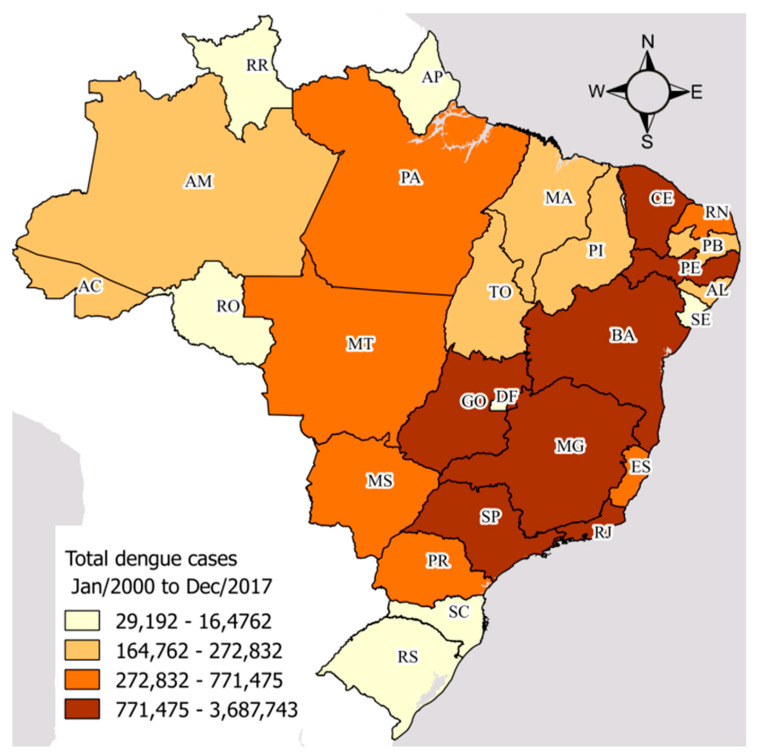
Distribution of total dengue cases from January 2000 to December 2017 across the study sites: states and federal district of Brazil.

Cases of dengue fever from January 2000 to December 2017 were confirmed in the 26 states and federal district of Brazil (Figure 1). The region-wise states studied were as follows. North: Acre (AC), Amazonas (AM), Amapá (AP), Pará (PA), Rondônia (RO), Roraima (RR), and Tocantins (TO); Northeast: Alagoas (AL), Bahia (BA), Ceará (CE), Maranhão (MA), Paraíba (PB), Pernambuco (PE), Piauco (PI), Rio Grande do Norte (RN), and Sergipe (SE); Central-West: Goiás (GO), Mato Grosso do Sul (MS), and Mato Grosso (MT); Southeast: Espírito Santo (ES), Minas Gerais (MG), São Paulo (SP), and Rio de Janeiro (RJ); South: Paraná (PR), Rio Grande do Sul (RS), and Santa Catarina (SC). The federal district (DF), which is in the state of Goiás, was also studied.

### 2.3. Data Source

The non-nominal database of confirmed monthly dengue cases from 2000 to 2017, without distinction by serotype, from the 26 states and the federal district were obtained from the information systems of the Health Surveillance Secretariat (Ministry of Health of Brazil). All dengue cases were confirmed through laboratory assays (serological methods, virus isolation, or antigen and/or nucleic acid detection) or, when occurring in the course of epidemic, they were confirmed by clinical-epidemiological protocols. Because of diagnostic challenges due to the unknown co-circulation of Zika and chikungunya viruses, a possible bias in the time-series is higher numbers of dengue cases from 2015 on than actually occurring [15]. The data requests were made via E-SIC through Law No. 12,527/2011, known as the Law of Access to Information (Electronic System of the Citizen Information Service) of the Federal Government (request protocol No. 25820003166201892). The database and scripts used in the analyses are available in the following online repository: https://github.com/MVMLima/Doutorado.

### 2.4. Variables

The following three discrete quantitative variables, which were transformed into natural logarithms, were used [28]: (1) number of monthly dengue cases from January 2000 to December 2016: this variable was used to adjust the parameters of the statistical models; (2) estimated values from January to December 2017 for each statistical model: forecasting variable; and (3) number of monthly dengue cases from January to December 2017: test variable, which was compared with the forecasting variable to assess the predictive capacity of the statistical models. The use of the natural log transformation was recommended to increase model performance in forecasting [28].

### 2.5. Data Analysis and Statistical Modeling

The employed statistical approach was based on time-series models. The first procedure was to test whether the time series was stationary by means of the Dikey-Fuller test, which was increased to a significance level of 5%. An assumption of stationariness was made in the use of the statistical models.

The time series covered 216 months from January 2000 to December 2017, which were divided into two periods: (1) training period from January 2000 to December 2016; (2) test period from January to December 2017. The number of monthly dengue cases during the training period was used to adjust each of the statistical models and to estimate the parameters of the temporal components (seasonality, linear trend, and stochastic effect). The number of monthly dengue cases in the test period was utilized for comparison with the values estimated by the statistical models. The test was performed using the three below-mentioned forecasting horizons: (1) 12 months in advance (January–December 2017); (2) 6 months in advance (July–December 2017) and (3) 3 months in advance (October–December 2017). The result of each test was used to evaluate the predictive accuracy of the models.

The statistical models used were as follows: (deterministic models) (1) ARIMA [21], (2) ETS [22], (3) TBATS [23], (4) BATS [23], and (5) STLM [24,29]; (stochastic models) (6) StructTS [25], (7) NNETAR [21], (8) ELM [30], and (9) MLP [31]; (10) null model [27].

Three criteria were considered to assess the predictive accuracy of the statistical models:

(1) Mean absolute percentage error (*MAPE*), in which the absolute difference (*A_t_* − *F_t_*) represents the distance between the actual value *A_t_* and the estimated value *F_t_* in the forecast. The ratio of the distance (*A_t_* − *F_t_*) to the actual value *A_t_* was multiplied by 100% to obtain the percentage distance. The sum of the percentage error calculated for each month of the time series was divided by the number of months to obtain the average percentage distance according to the formula given below:
  (1) $$MAPE=\frac{100\%}{n}\overset{n}{\underset{t=1}{\sum }}\left|\frac{A_{t}-F_{t}}{A_{t}}\right|$$
(2) Relative *MAPE* scale, in which the *MAPE* of the null model is divided by the *MAPE* value of each model. If the result of this division is ≤1, the model is classified as having poor predictive accuracy. If the value is >1 and ≤2, the model is classified as having low predictive accuracy. A relative *MAPE* value >2 means that the model possesses reliable predictive accuracy.
(3) Coefficient of uncertainty (Theil’s U) measures the relative accuracy by penalizing statistical models with high deviations from the mean value. Values <1 represent reliable predictive capability [27].

Acceptable predictive capacity was defined as a combination of three criteria in specific results: (1) low *MAPE* value, (2) values >2 for the relative *MAPE* scale, and (3) values <1 for Theil’s U.

The analyses were performed through the computational environment of book access using programming *scripts* that can be reproduced by surveillance teams of the municipalities and states [32]. These scripts have been made available in the online repository (see Data Source).

## 3. Results

Totally, 16 million (16,654,340) dengue cases were confirmed in the states and federal district of Brazil from 2000 to 2017 (Table 1), with an average of 77,000 (±108,341) cases per month.

**Figure 2:**
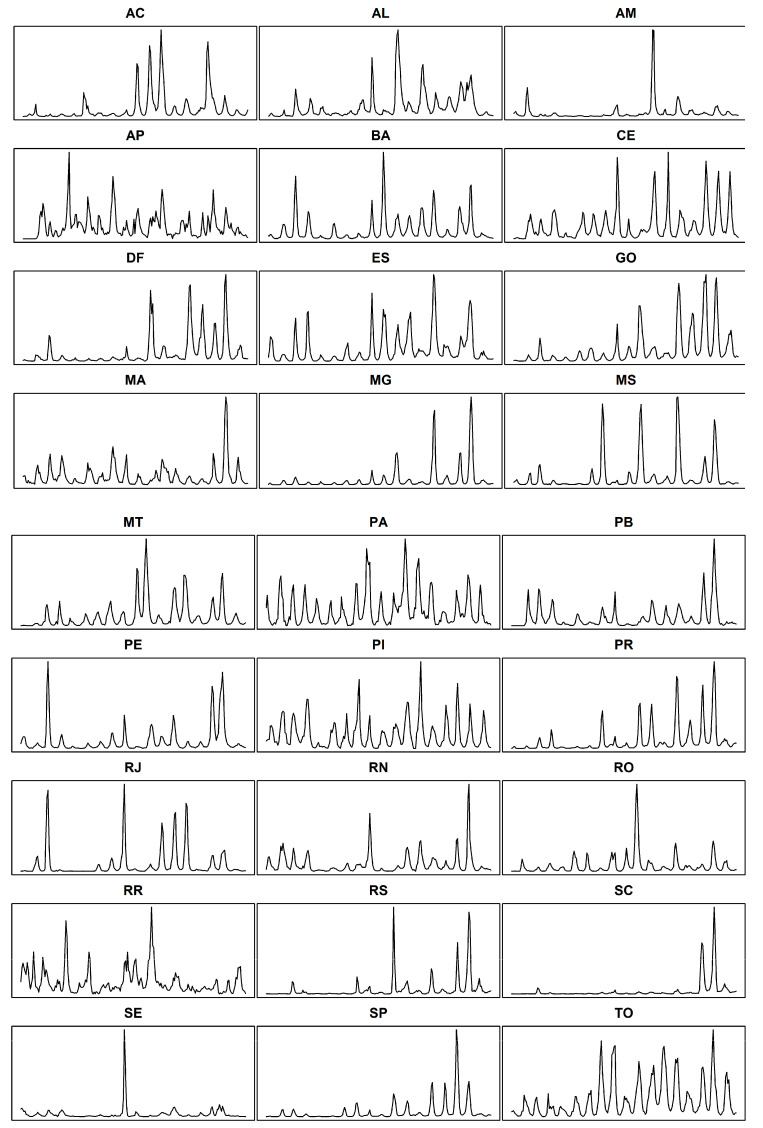
Time series of dengue cases in the states and federal district of Brazil from January 2000 to December 2017. *Y*-axis: number of monthly-dengue cases. *X*-axis: time period (January/2000 to December/2017).

The time series of dengue cases in the states and federal district of Brazil showed steadiness (Table 1) and the presence of a seasonal pattern of the disease (Figure 2).

The deterministic models (ARIMA, ETS, BATS, TBATS, and STLM) were reliable for predicting dengue in six states and the federal district (AC, CE, DF, MA, MT, PA, and PI) in the 12-month forecasting horizon (Table 2). These models were also reliable for predicting dengue in seven states (AC, AP, CE, MA, PI, PR, and SE) in the 6-month forecasting horizon. The stochastic models (NNETAR, StructTS, ELM, and MLP) were reliable in two states (BA and RN) in the 6-month forecasting horizon and in eight states (AM, BA, ES, GO, PI, PR, RN, and RO) in the 3-month forecasting horizon (Table 2). 

**Figure 3:**
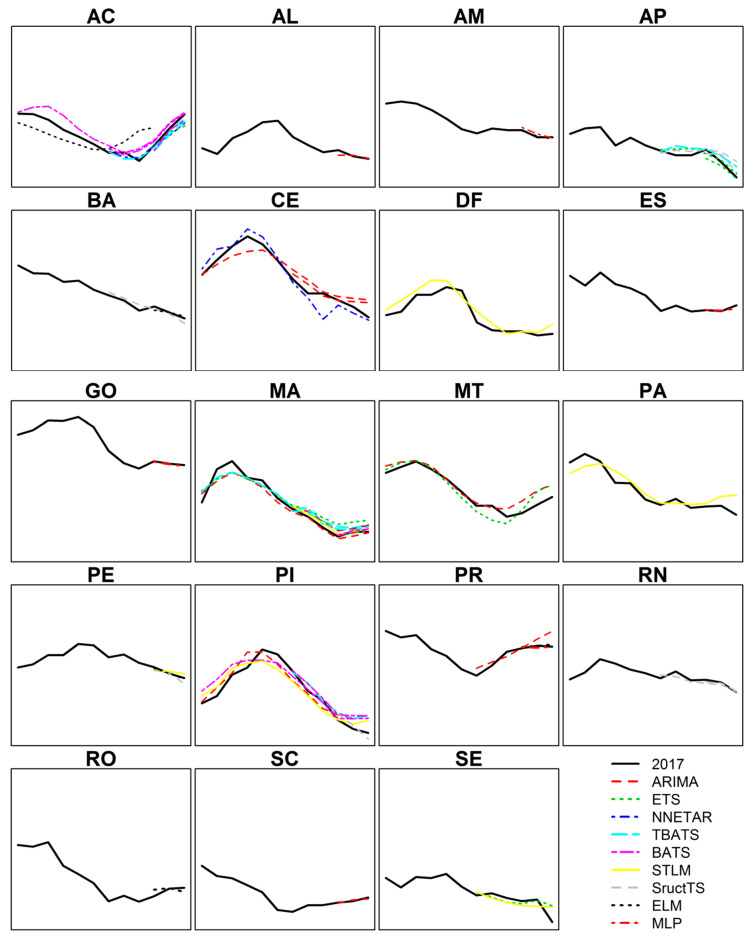
Time series of dengue cases (log-*n*) and the prediction of each model considered reliable in the states and federal district of Brazil from January 2017 to December 2017. *Y*-axis: natural logarithm of the number of monthly-dengue cases. *X*-axis: time period (January–December 2017).

Eighteen states (70%) and the federal district presented reliable models for predicting dengue in at least one of the forecasting horizons (Figure 3). Three states (AC, MA, and PI) presented reliable models for predicting dengue in all three horizons (Supplementary Material—Text S2). Eight states (MG, MS, PB, RJ, RR, RS, SP, and TO) did not present any reliable model for predicting dengue. Full data and results are presented in Supplementary Material—Text S3.

## 4. Discussion

The models used here were adjusted with the training time series from January 2000 to December 2016 and were tested against the actual data from the January–December 2017 time series in the three forecasting horizons. The first interpretation of the results is that we did not find a foolproof model that was capable of making reliable predictions for all the forecasting horizons and for all the states and the federal district of Brazil. It can also be stated that the deterministic models (ARIMA, ETS, BATS, TBATS, and STLM) reliably predicted dengue in the 12-month forecasting horizon for the states of AC, CE, MA, MT, PA, and PI and the federal district. The stochastic models (NNETAR, StructTS, MLP, and ELM) were reliable in the 3-month forecasting horizon for the states of AM, AP, BA, ES, GO, PE, PI, PR, RN, and RO. This difference in predictive performance between the deterministic and stochastic models could be attributed to the seasonal pattern of the disease, which was observed over the 12-month forecasting horizon. Deterministic models have predictive capacity to extrapolate the seasonal pattern of the disease and should therefore be used for long-term forecasting horizons such as 12 months. Stochastic models are more appropriate for short-term applications, such as 3 months, during which the stochastic component predominates over seasonality. Finally, we would like to highlight that these models were reliable in predicting dengue in the three states of AC, MA, and PI for all the forecasting horizons. Therefore, we strongly recommend incorporating the work routine of this study in the dengue surveillance activities of these states.

We observed that forecasting the future scenarios of disease distribution in the population enables decision making and planning to reduce the societal burden [33]. Time-series analysis tools similar to those investigated in this study have been widely advocated by several authors to predict the occurrence of infectious diseases such as dengue fever [34]. The magnitude of the “dengue problem” can be understood from the fact that the number of cases reported in the Americas in 2019 was 3,139,335. This is the highest figure ever recorded and is 30% higher than the number of cases reported during the 2015–2016 epidemic. In Brazil, 2,226,865 probable cases of dengue fever were reported in 2019, including 789 deaths. Of these, 1,244,082 were confirmed in the laboratory, among which 19,187 were classified as dengue with warning signs and 1453 as severe dengue [35]. These figures demonstrate the pressing need to develop models capable of predicting the incidence of the disease [19,20,23,30,36].

The use of time series of dengue cases without resorting to other exogenous variables such as environmental or socioeconomic variables [37,38] proved to be a strategy that maximizes the predictive capacity of the statistical models [39]. Two deterministic statistical models that performed exceptionally well were the ARIMA and TBATS, which were considered reliable for detecting dengue seasonality in other studies too [40]. The TBATS model was also able to accurately describe the behavior of other seasonally-distributed infectious diseases in England and Wales [41]. Although several approaches exist for dengue prediction (e.g., [42]), the ARIMA model is preferred [43] because it is more reliable and easier to interpret than other approaches based on fuzzy [44] or Bayesian logic [45,46]. It is inferred that the use of this statistical model will allow surveillance services to effectively predict the number of dengue cases, thereby providing a useful overview of the scenario to decision-makers.

Stochastic models constitute the more recent approaches to dengue forecasting. Baqueiro et al. [47] comprehensively compared deterministic (ARIMA) and stochastic models (neural networks) for predicting dengue with a forecasting horizon of 1 month in the city of São Paulo. Storlerman et al. [48] employed machine learning algorithms to detect climatic signatures that correlated with the total number of dengue cases in some Brazilian capitals. Guo et al. [49] developed dengue prediction models using artificial intelligence algorithms for Guangdong, China. The stochastic ELM model was applied in the present study and presented reliable performance in the 3-month forecasting horizon for the states AM, BA, PE, PR, and RO. This result is in agreement with the study by Baqueiro et al. (2018) [47], which predicted dengue for a 1-month forecasting horizon.

The health services in the Brazilian states today possess digitized disease data in the integrated online system (SINAN), which can be fed into statistical models such as the ones used in the present study. We recommend the use of these models for state surveillance, especially in AC, MA, and PI, combined with the epidemiological data available in SINAN [50]. External variables (climate and social environment) [51] are also important; however, the use of the SINAN data with the statistical models presented here is totally feasible within the current structure of the state health services. Additionally, there is a spatiotemporal correlation between incidence of symptomatic cases and the presence of asymptomatic reservoirs in dengue transmission areas [52].

The failure to consider the external factors (climatic, environmental, social, or immunological) [53] and the building infestation index [54] could be viewed as a possible limitation of this study.

## 5. Conclusions

We identified statistical models (ARIMA, TBATS, and ELM) that can be used to predict dengue in Brazilian states. If these models are applied by state health services, dengue epidemics could be effectively predicted and their impact reduced through interventions such as vector control and preparation of health systems to handle a large number of patients.

Dengue remains to be one of the most important global public health challenges. The possibility of using a free tool to predict the emergence of epidemics can make a huge difference, since it enables health services to offer enhanced assistance to the population. Therefore, we reinforce that the most salient role of forecasting models in epidemiological studies is to aid in decision making for health infrastructure planning to meet the needs of the population.

## Figures and Tables

**Table 1:** Descriptive statistics of the number of dengue cases confirmed according to the states and federal district of Brazil from 2000 to 2017.

Brazilian States and the Federal District	Total Jan. 2000–Dec. 2017	Monthly Average (Standard Deviation) *n* = 216	Median Monthly *n* = 216	Minimum Monthly *n* = 216	Maximum Monthly *n* = 216	Test of Stationariness *n* = 216
Acre	209,830	971 (1764)	323	6	10,653	Yes
Alagoas	272,832	1263 (1704)	611	22	10,818	Yes
Amazonas	210,506	975 (2395)	437	13	22,319	Yes
Amapá	58,500	271 (264)	187	0	1881	Yes
Bahia	894,494	4141 (6005)	1632	67	41,277	Yes
Ceará	975,734	4517 (5414)	2525	66	29,665	Yes
Distrito Federal	141,632	656 (1179)	216	7	6802	Yes
Espírito Santo	560,814	2596 (3365)	1329	67	18,937	Yes
Goiás	1,152,397	5335 (8135)	2191	51	40,608	Yes
Maranhão	168,909	782 (1018)	470	28	7986	Yes
Minas Gerais	2,385,230	11,043 (27,958)	2565	8	202,922	Yes
Mato Grosso do Sul	512,062	2371 (5033)	551	1	27,348	Yes
Mato Grosso	393,688	1823 (2584)	828	0	16,566	Yes
Pará	303,257	1404 (1270)	890	225	6812	Yes
Paraíba	235,798	1092 (1616)	470	0	12,189	Yes
Pernambuco	822,514	3808 (6554)	1442	193	41,966	Yes
Piauí	167,531	776 (782)	481	21	4714	Yes
Paraná	720,436	3335 (6170)	1164	15	37,914	Yes
Rio de Janeiro	1,696,598	7855 (16,823)	1684	32	100,762	Yes
Rio Grande do Norte	400,889	1856 (2665)	928	71	21,847	Yes
Rondônia	161,992	750 (1322)	336	9	11,556	Yes
Roraima	84,386	391 (358)	269	53	2480	Yes
Rio Grande do Sul	29,192	135 (339)	34	1	2621	Yes
Santa Catarina	35,900	166 (532)	35	2	4768	Yes
Sergipe	125,160	579 (1431)	233	22	17,612	Yes
São Paulo	3,687,743	17,073 (37,676)	4976	262	322,982	Yes
Tocantins	246,316	1140 (1194)	699	29	5730	Yes

**Table 2:** Reliable statistical models for predicting dengue cases in three forecasting horizons according to the states and federal district of Brazil from 2000 to 2017.

Brazilian States and the Federal District	Reliable Models (12-Month Forecasting Horizon)	Reliable Models (6-Month Forecasting Horizon)	Reliable Models (3-Month Forecasting Horizon)
Acre	ELM, BATS	TBATS, NNETAR, BATS	BATS, TBATS, ETS
Alagoas	-	-	ARIMA
Amazonas	-	-	ELM, MLP
Amapá	-	ETS, TBATS, STLM	ETS, StructTS
Bahia	-	StructTS	ELM
Ceará	NNETAR, ARIMA	ARIMA	-
Distrito Federal	STLM	-	-
Espírito Santo	-	-	MLP
Goiás	-	-	MLP
Maranhão	ARIMA, TBATS, ETS	STLM, TBATS, ARIMA	ETS, BATS, ARIMA
Minas Gerais	-	-	-
Mato Grosso do Sul	-	-	-
Mato Grosso	ARIMA, ETS	-	-
Pará	STLM	-	-
Paraíba	-	-	-
Pernambuco	-	-	ELM, StrucTS, STLM
Piauí	ARIMA, STLM, BATS	TBATS, BATS	StructTS
Paraná	-	ARIMA	ELM, MLP
Rio de Janeiro	-	-	-
Rio Grande do Norte	-	StructTS	StructTS
Rondônia	-	-	ELM
Roraima	-	-	-
Rio Grande do Sul	-	-	-
Santa Catarina	-	-	ARIMA
Sergipe	-	ETS, STLM	-
São Paulo	-	-	-
Tocantins	-	-	-

## Supplementary Materials

Supplementary Materials are available online at https://www.mdpi.com/2075-4450/11/11/794/s1, Text S1: Detailed information and rationale on each of the selected models for this study is herein presented; Text S2: A more detailed description of results from specific states (AC, MA, PI) is shown; Text S3: Full data and complete results are presented.
