# Supplementary material for: Evaluation of the Models for Forecasting Dengue in Brazil from 2000 to 2017: An Ecological Time-Series Study
Source: Insects. 2020 Nov 12;11(11):794. doi: 10.3390/insects11110794 (PMC7696623; doi:10.3390/insects11110794)
Supplement: Supplementary file 1 [file insects-11-00794-s001.zip › insects-977476-supple - conversion/insects-977476 - Supple - Text S1.docx]

**Supplementary material – Text S1:** Detailed information and rationale on each of the selected models for this study is herein presented. The main assumption for model selection was having all selected models in a single, open-software analytic environment in order to help in the implementation of these tools for the state health secretaries in Brazil. Additionally, a mixture of deterministic in combination with stochastic models was preferred to build a diverse set of models for forecasting dengue epidemics in Brazil. The models are fully described with the equations and rationale for fitting time series, as follows.

**ARIMA**

Originally proposed by Box and Jenkins in the 1970s, the autoregressive integrated moving average (ARIMA) model has become widely-known for its simplicity and flexible statistical structure for the study and forecast of time series [1]. It is composed by three parameters: AR, autoregressive parameter – utilized for estimating or considering the temporal autocorrelation of the response-variable (eg, dengue cases); I, integrative parameter – applied when time series shows non-stationarity and needs to be adjusted for a better fit; and MA, moving average parameter – used for depicting cyclic or linear trends in the regression error over time [1]. As an example, let’s consider a stationary process of dengue cases over time to be adjusted by a model specified with one AR term and one MA term as follows:

$$Y_{t}= \alpha+\beta_{1}Y_{t-1}+{\phi_{1}\epsilon}_{t-1}$$

which means that the predicted number of dengue cases (*Y_t_*) is a function of a constant (α) plus a linear combination lag of dengue cases (*β_1_Y_t-1_*) plus a linear combination of lagged forecast error (*φ_1_ϵ_t-1_*). This model is formally denoted as ARIMA (1,0,1), whereas another model with two AR, one I, and three MA terms will be denoted as ARIMA(2,1,3). In the *R* programming environment (R Development Core Team), a function named as *auto.arima* is available in the package *forecast* [1]. This function was herein applied because it automatically selects the most appropriate ARIMA model specification to the available time series data.

**ETS**

Exponential smoothing refers to a class of time series forecasting methods. In particular, ETS stands for the following model specification terms: (1) error (E) – the unpredictable component, (2) trend (T) – pattern that repeats itself with known periodicity, and (3) seasonal (S) – the long-term direction. The terms can be combined in various ways, such as a purely additive model:

$$y=T+S+E$$

where the number of dengue cases (*y*) is a function of additive effects of trend (*T*), seasonal (*S*), and error (*E*) components. On the other hand, a purely multiplicative model would be specified as:

$$y=T*S*E$$

where *y* is in function of multiplicative effects of the model terms (E,T,S). Trend and seasonal components are further decomposed in other terms. For instance, let’s illustrate this with the trend (T). This component is decomposed into a constant (α) and a growth term (*β*). Depending on how α and *β* are combined, it is possible to have five trend types:

$$None: T_{h}= \alpha$$

$$Additive: T_{h}=\alpha+\beta_{h}$$

$$Additive damped: T_{h}=\alpha+(\phi+\phi^{2}+\ldots+\phi^{h})\beta$$

$$Multiplicative: T_{h}=\alpha\beta^{h}$$

$$Multiplicative damped: T_{h}=\alpha\beta^{(\phi+\phi^{2}+\ldots+\phi^{h})}$$

where *T_h_* is the forecast trend over the next *h* time periods and *φ* is a damping term (0 < *φ* < 1) that will dampen the effect of *β* as time increases. Supposing a trend component is chosen, then seasonal and error components will be additively or multiplicatively selected. This illustrates how flexible ETS models can be for forecasting time series. A programming function written in R programming environment (R Development Core Team) named as *ets* was herein utilized from the package *forecast* [1]. This function automatically selects the best ETS model specification conditioned to the time series data [2].

**BATS and TBATS**

These are exponential smoothing methods that represent further innovations to the ETS method. BATS model stands for (1) B, Box-Cox transformation for dealing with non-linear data; (2) A, ARMA model for residuals; (3) T, Trend component; and (4) S, Seasonal component. BATS model was shown to improve the prediction performance in comparison with ETS model [3]. When seasonality is complex and highly frequent, however, BATS model can fail. A further improvement was proposed [4]. This helped in the creation of TBATS model, which is a BATS model with a trigonometric seasonal algorithm for dealing with seasonally-complex time series [4]. For the sake of simplicity, equations for BATS and TBATS models were omitted – readers can check them elsewhere in [3] and [4]. Functions in R programming language *bats* and *tbats* from the package *forecast* [1] were applied herein with each time series of dengue cases. These functions have optionally parallel processing to speed up computations [1] of the model components described in [3] and [4].

**STLM**

This method is relative to ETS model in a different way that BATS and TBATS are. While the latter models increase model complexity with rather complex algorithms, the Seasonal and Trend decomposition using Loess model (STLM) simplifies the workload for ETS model. As an illustration, let’s suppose a seasonally-complex time series. A seasonal adjustment is first performed via Cleveland-style loess method [5]. The seasonally-simplified or non-seasonally time series is then adjusted by ETS model with trend and error components only. Lastly, previous outcome is then re-seasonalised to obtain the final forecast [1]. STLM is available in R programming language as *stlm* function in the *forecast* package [1].

**StructTS**

Structural time series model (StructTS) is composed by building blocks that define seasonal and trend components. Unlike previous models (ARIMA, ETS, BATS, TBATS, and STLM), the optimization procedures of StructTS is much more complex. Sometimes it is not possible to replicate results, because each model run leads to a different result – this may happen because optimization algorithms can converge to different local best estimates. The usual procedure to fit StructTS to a time series is as follows: (1) choose arbitrary starting values for parameters, (2) evaluate the log-likelihood function, (3) obtain a new set of parameter values by optimization algorithms, and (4) iterate the searching procedure until a predetermined degree of convergence [6]. The basic structural model is defined as follows:

$$y_{t}= \mu_{t}+\gamma_{t}+\epsilon_{t}, \epsilon_{t} \sim N(0,\sigma^{2})$$

where the predicted number of dengue cases (*y_t_*) is a function of random walk with deterministic drift (*µ_t_*) plus seasonal component with dynamics (*γ_t_*) plus normally and independently distributed error term (*ϵ_t_*). The R function *StructTS* available in the *forecast* package runs the abovementioned fitting procedures with the basic structural model to observed time series [1].

**NNETAR**

Neural Network Autoregression (NNETAR) model has recently been gaining attention due to its successful use for forecasting of time series data. Deterministic models, such as ARIMA, assume a linear relationship between inputs and outputs. NNETAR, however, can approximate any continuous function, including nonlinear functions. NNETAR can be illustrated as a network of nodes or “neurons” which are organized in layers. The predictors are in the input layer, while the forecasts are in the output layer. In-between there may also be intermediate layers containing “hidden neurons”. That is why NNETAR is seen as a black-box – these hidden neurons in the intermediate layer can make a big difference to the forecasts (Figure 1 – Text S1).


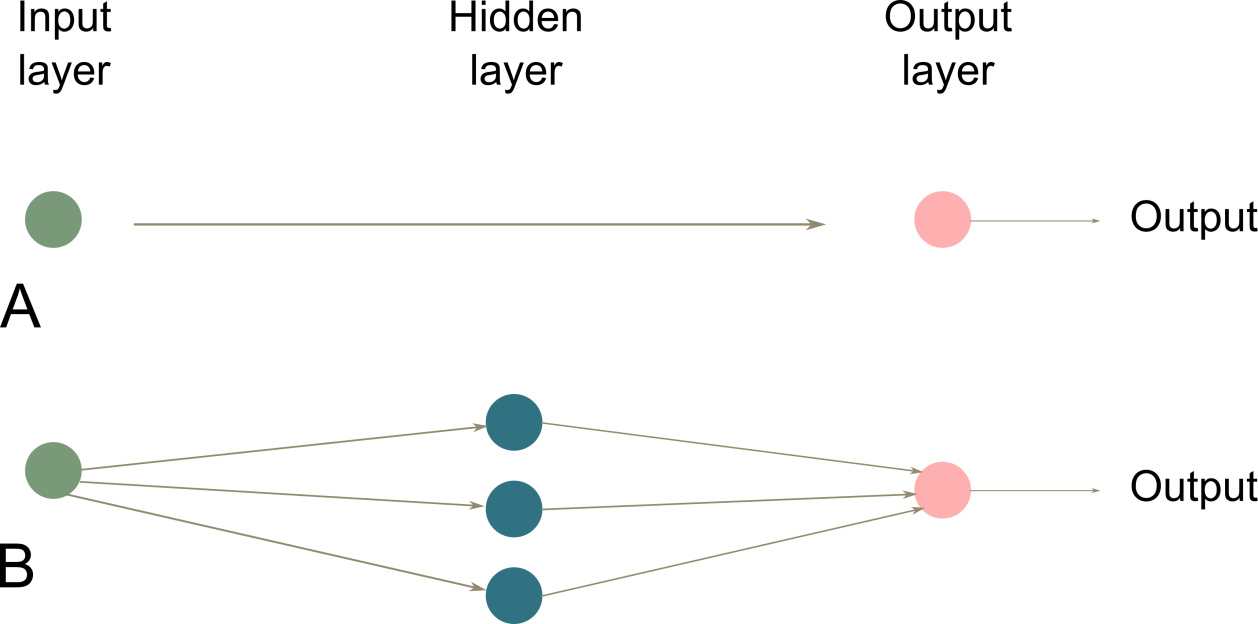


**Figure 1 – Text S1**. Illustrative panels of the inner structure of neural networks in two scenarios: (**A**) in its simplest form, there is no hidden layer and the neural network is equivalent to a linear method such as ARIMA; (**B**) with the addition of one intermediate layer, the neural network becomes non-linear.

In the case (B) depicted in Figure 1 – Text S1, this is known as a feed-forward neural network with a single hidden layer. This illustrative panel B is exactly what Hyndman and Caceres developed in the R function *nnetar* in the package forecast [1]. On one hand, one can say that this is a very “simple” neural network because it has one hidden layer only. One the other hand, the addition of a single hidden layer can increase model complexity with non-linear functions. The predictors in the input layer are weighted by these non-linear functions and a “learning algorithm” that minimizes the “cost function” to generate forecasts in the output layer. The reader should understand “learning algorithm” as one out multiple choices in the now widespread field of machine learning. Lagged values of the time series can be used as inputs as well – so this makes this neural network model as autoregressive or, simply known as NNETAR.

**ELM** and **MLP**

These are more complex methods of neural networks than NNETAR – multiples hidden layers can be selected and the “learning algorithm” is more powerful [7,8]. Extreme learning machines (ELM) and Multilayer perceptron (MLP) methods for time series forecasting are available as R functions *elm* and *mlp* in the package *nnfor* [9]. These functions were herein applied with the default options in which the number of hidden layers is automatically selected.

**Naïve**

This is the null model [10]. Its forecast is a constant value over time of forecasting horizon. This constant value equals the last observed value in the studied time series [10]. If its nickname “naïve” is appropriately said or not, it can be a matter of debate; however, it is definitely deceiving because this model sometimes outperforms the “big guys” above in well-behaved and non-seasonal time series.

**References**

[1] Hyndman, R.J.; Khandakar, Y. Automatic time series forecasting: The forecast package for R. *J. Stat. Softw.* **2008**, 27, C3.

[2] Hyndman, J.R.; Anne, K.B.; Ralph, S.D.; Simone, G. A state space framework for automatic forecasting using exponential smoothing methods. *Int. J. Forecast* **2002**, 18, 439–54.

[3] De Livera, A.M. *Automatic forecasting with a modified exponential smoothing state space framework*. Australia, Monash Econometrics and Business Statistics Working Papers 10, 2010.

[4] de Livera, A.M.; Hyndman, R.J.; Snyder, R.D. Forecasting time series with complex seasonal patterns using exponential smoothing. *J. Am. Stat. Assoc.* **2011**, 106, 1513–1527.

[5] Cleveland, R.; Cleveland, W.; McRae, J.; Terpenning, I. STL: a seasonal-trend decomposition procedure based on Loess (with discussion). *J. Off. Stat.* **1990**, 6, 3.

[6] Durbin, J.; Koopman, S.J. *Time Series Analysis by State Space Methods*. Oxford, Oxford University Press, Cary, NC, USA, 2013.

[7] Crone, S.F.; Kourentzes, N. Feature selection for time series prediction—A combined filter and wrapper approach for neural networks. *Neurocomputing* **2010**, 73, 1923–1936.

[8] Kourentzes, N.; Barrow, D.K.; Crone, S.F. Neural network ensemble operators for time series forecasting. *Expert. Syst. Appl*. **2014**, 41, 4235–44.

[9] Kourentzes, N. *nnfor: Time Series Forecasting with Neural Networks*. R package version 0.9.6., 2019. Available online: <https://CRAN.R-project.org/package=nnfor> (accessed on 30 October 2020)

[10] Hyndman, R.J.; Athanasopoulos, G. *Forecasting: Principles and Practice*. 2nd ed. Melbourne, Australia: OTexts; 2018. 382 p.

|  |  |
| --- | --- |
