# Supplementary material for: Evaluation of the Models for Forecasting Dengue in Brazil from 2000 to 2017: An Ecological Time-Series Study
Source: Insects. 2020 Nov 12;11(11):794. doi: 10.3390/insects11110794 (PMC7696623; doi:10.3390/insects11110794)
Supplement: Supplementary file 1 [file insects-11-00794-s001.zip › insects-977476-supple - conversion/insects-977476 - Supple - Text S2.docx]

**Supplementary material – Text S2:** A more detailed description of results from specific states (AC, MA, PI) is shown. In these states the proposed analytical approach showed the most successful outcomes with accurate forecasting of dengue cases.

**Detailed description of results from specific states**

Time series of number of monthly-dengue cases in the states of Acre (AC), Maranhão (MA), and Piauí (PI) are shown below (Figure 1 – Text S2),


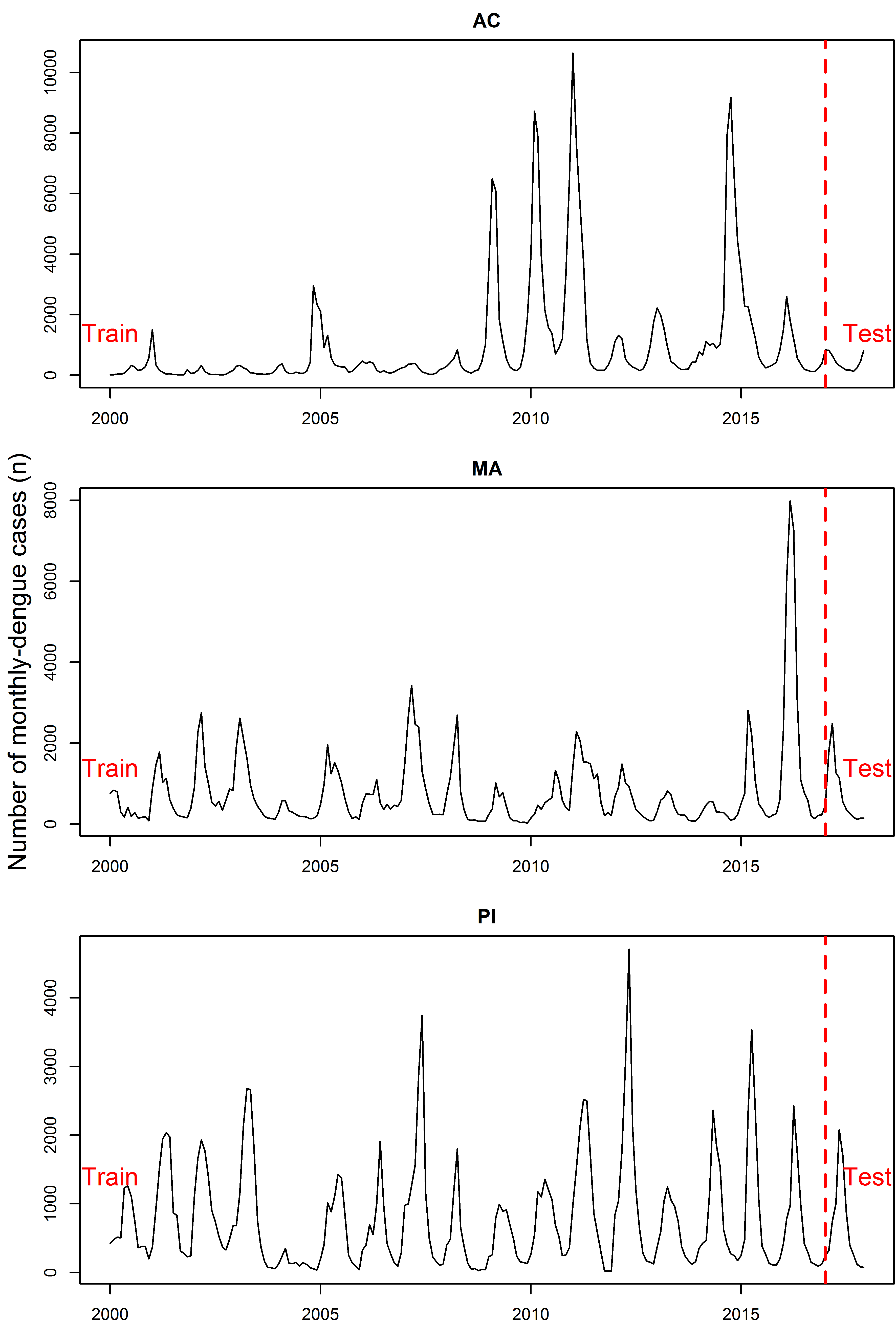


**Figure 1 – Text S2**. Time series herein studied.

These time series were split in two segments: (1) train segment (January 2000 to December 2016) and (2) test segment (January to December 2017) (Figure 1 – TextS2). The train segment was the input for each model. Each model’s output was compared with the test segment. When the error between output and test segment was lower than the predetermined threshold of model acceptable performance (see the main text), the associated forecast was considered accurate. This occurred in all forecasting horizons in all of these states. In Acre state it is possible to observe BATS model “hunting” the test segment (black solid line) in panels A, B, and C (Figure 2 – Text S2).


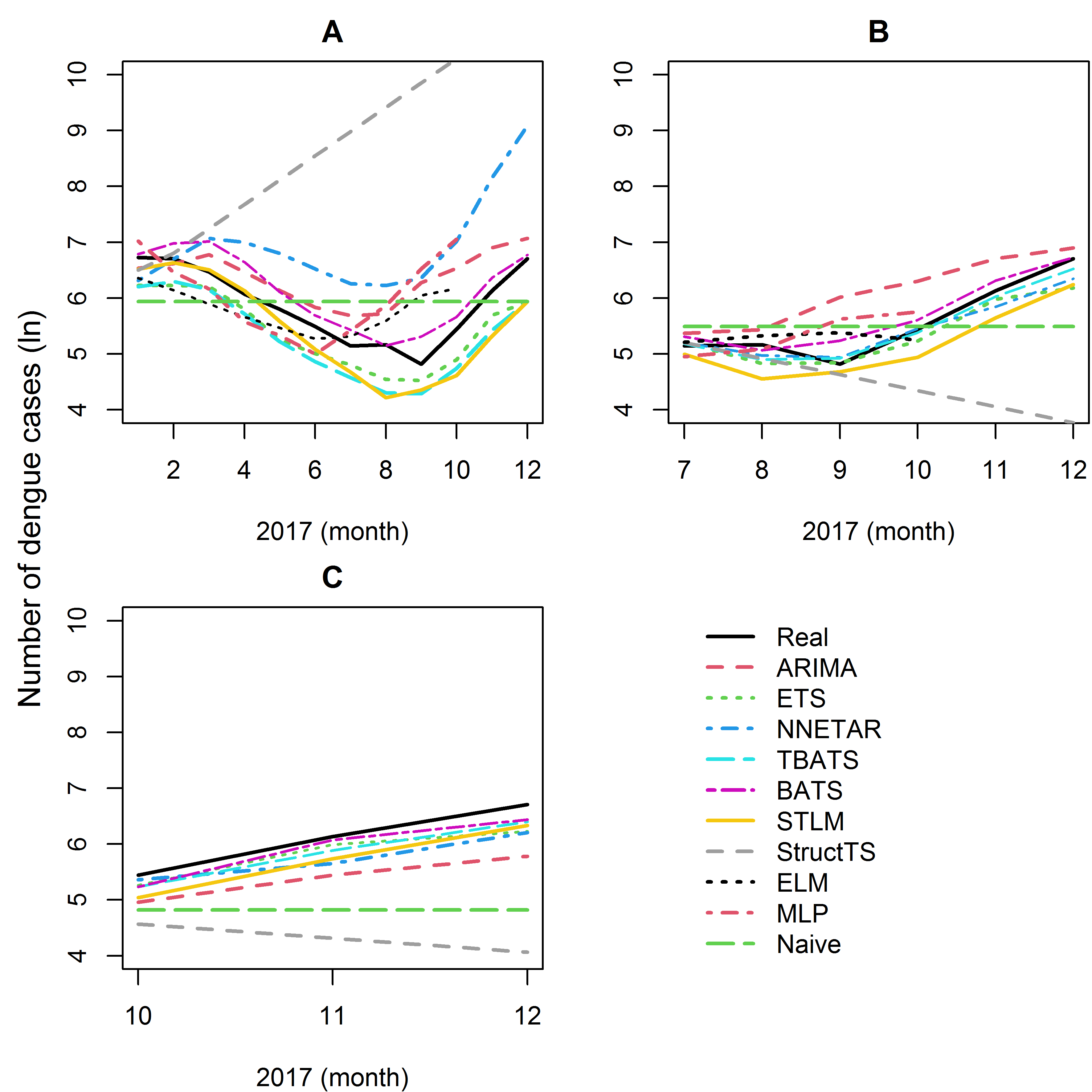


**Figure 2 – Text S2**. Graphical comparison of each model in relation to the test segment (black solid line) – if a given model prediction (BATS – magenta two-dashed line) is following the test segment over the forecasting horizon, the interpretation is straightforward: this can be a reliable model for forecasting dengue cases in Acre state. (**A**) 12-month forecasting horizon. (**B**) 6-month forecasting horizon. (**C**) 3-month forecasting horizon.

The same approach was carried out in the other states and the federal district. In Piauí state (PI) the test segment (black solid line) was followed by ARIMA (red dashed line) and other deterministic models (ETS, green dotted line), while it was completely missed by stochastic models as NNETAR (blue dot-dashed line) or ELM (black dotted line) in the 12-month forecasting horizon (Figure 3 – Text S2 – panel A). In the 6-month forecasting horizon all models are following the test segment, except Naïve (green long-dashed line) and StructTS (gray dashed line) (Figure 3 – Text S2 – panel B); in contrast and ironically, StructTS (gray dashed line) seems the best “chaser” to the test segment in the 3-month forecasting horizon (Figure 3 – Text S2 – panel C). In agreement with this, results from the state of Maranhão show a mixture of the same abovementioned patterns and explanations (Figure 4 – Text S2).


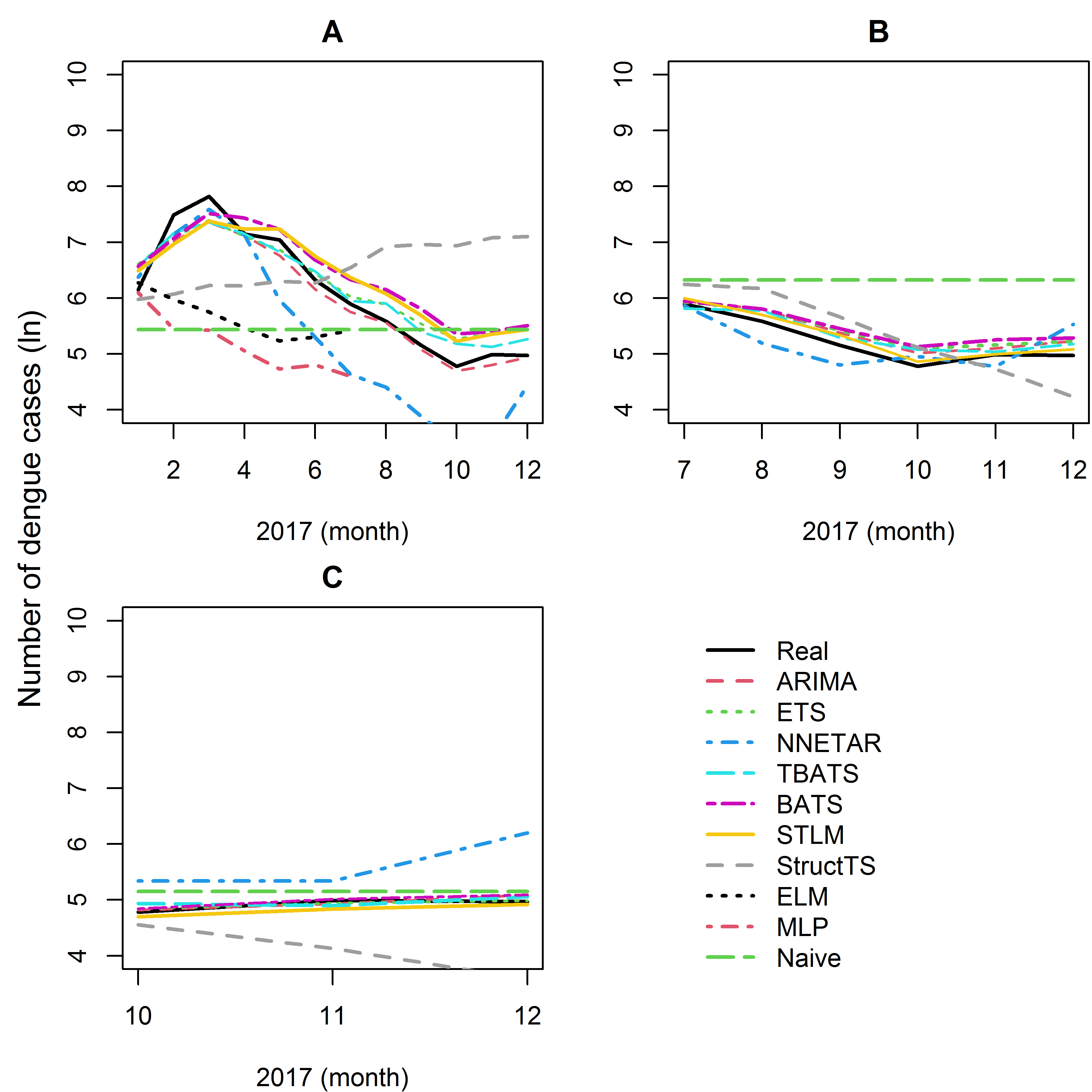


**Figure 3 – Text S2**. Graphical comparison of each model in relation to the test segment (black solid line) in the state of Piauí in each of the forecasting horizons (**A**) 12-month, (**B**) 6-month, and (**C**) 3-month.


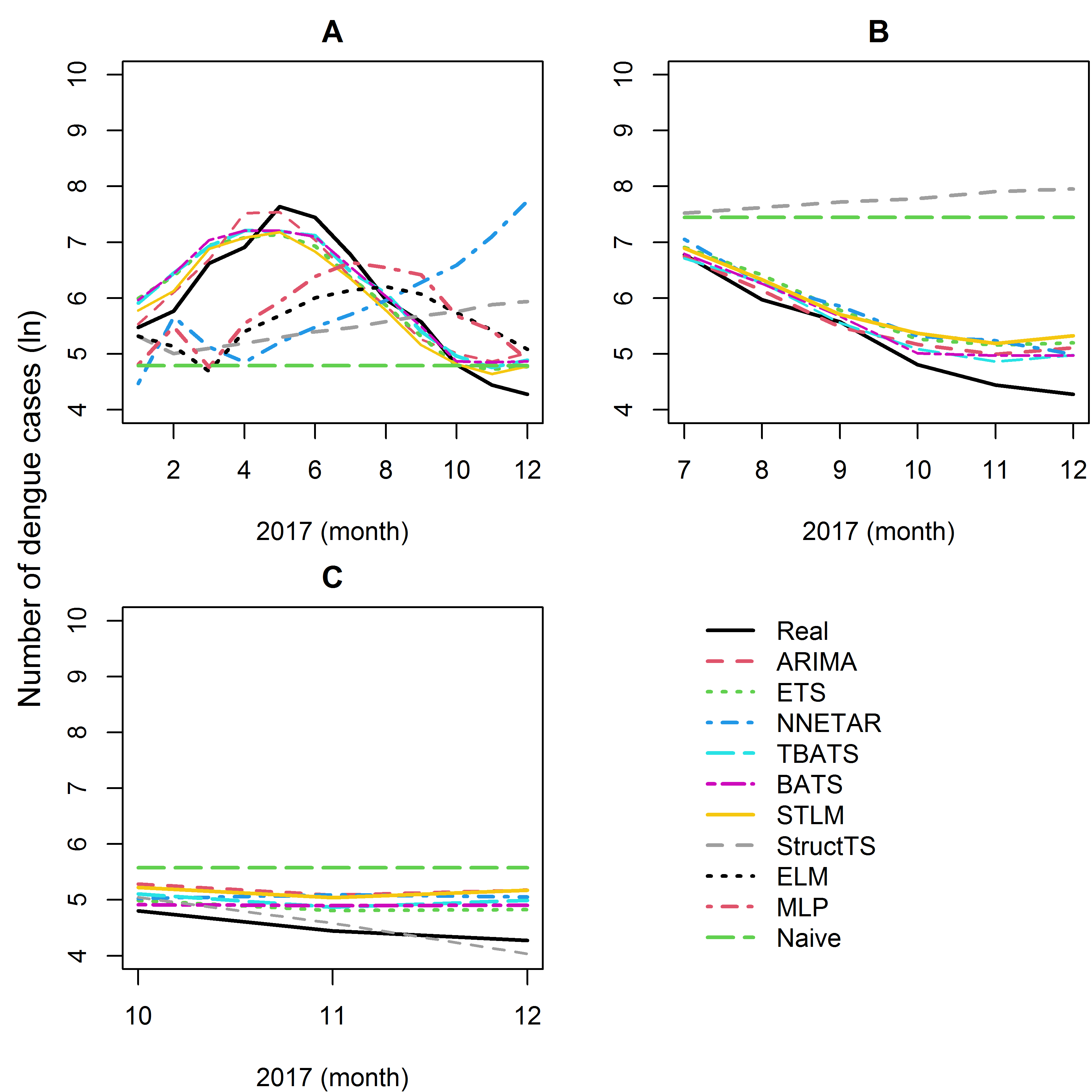


**Figure 4 – Text S2**. Graphical comparison of each model in relation to the test segment (black solid line) in the state of Maranhão in each of the forecasting horizons (**A**) 12-month, (**B**) 6-month, and (**C**) 3-month.
