# Supplementary material for: Evaluation of the Models for Forecasting Dengue in Brazil from 2000 to 2017: An Ecological Time-Series Study
Source: Insects. 2020 Nov 12;11(11):794. doi: 10.3390/insects11110794 (PMC7696623; doi:10.3390/insects11110794)
Supplement: Supplementary file 1 [file insects-11-00794-s001.zip › insects-977476-supple - conversion/insects-977476 - Supple - Text S3.docx]

**Supplementary material – Text S3:** Full data (Table 1–Text S3) and complete results (Tables 2–4– Text S3) are presented as follows.

**Table 1 – Text S3.** Number of confirmed dengue cases per year and state in Brazil, 2000 a 2017.

| **State** | **2000** | **2001** | **2002** | **2003** | **2004** | **2005** | **2006** | **2007** | **2008** | **2009** | **2010** | **2011** | **2012** | **2013** | **2014** | **2015** | **2016** | **2017** |
| --- | --- | --- | --- | --- | --- | --- | --- | --- | --- | --- | --- | --- | --- | --- | --- | --- | --- | --- |
| AC | 2,118 | 2,482 | 1,094 | 1,550 | 7,001 | 6,918 | 2,781 | 2,348 | 4,542 | 23,032 | 42,194 | 30,841 | 8,473 | 9,194 | 36,715 | 14,059 | 9,209 | 5,279 |
| AL | 1,580 | 2,394 | 11,973 | 9,989 | 6,602 | 3,741 | 4,735 | 13,002 | 19,578 | 6,068 | 54,378 | 11,102 | 33,686 | 15,665 | 16,499 | 33,859 | 24,475 | 3,506 |
| AM | 6,507 | 19,934 | 3,197 | 4,894 | 1,232 | 1,403 | 1,133 | 3,908 | 11,585 | 3,037 | 9,727 | 68,480 | 8,784 | 23,883 | 11,461 | 7,813 | 15,262 | 8,266 |
| AP | 0 | 3,846 | 1,688 | 5,933 | 3,891 | 3,994 | 2,732 | 5,687 | 2,298 | 3,041 | 4,440 | 4,281 | 2,375 | 2,290 | 2,624 | 4,407 | 3,536 | 1,437 |
| BA | 11,032 | 34,834 | 87,128 | 48,820 | 6,643 | 27,017 | 10,358 | 14,805 | 50,389 | 123,684 | 62,060 | 55,479 | 74,696 | 84,924 | 24,885 | 79,162 | 83,338 | 15,240 |
| CE | 2,410 | 46,000 | 33,234 | 52,448 | 10,362 | 46,995 | 43,792 | 51,375 | 84,725 | 20,603 | 34,751 | 90,917 | 72,757 | 56,003 | 42,046 | 105,614 | 100,439 | 81,263 |
| DF | 581 | 2,840 | 6,435 | 2,079 | 910 | 1,019 | 1,264 | 2,228 | 3,277 | 1,810 | 20,513 | 6,919 | 3,405 | 26,556 | 18,385 | 12,969 | 24,010 | 6,432 |
| ES | 20,734 | 8,598 | 28,150 | 32,684 | 5,392 | 6,520 | 15,469 | 10,451 | 39,897 | 51,706 | 42,075 | 54,594 | 23,880 | 82,956 | 25,869 | 44,840 | 54,722 | 12,277 |
| GO | 2,711 | 13,514 | 29,230 | 13,129 | 9,065 | 23,350 | 30,814 | 18,550 | 47,133 | 50,738 | 116,486 | 42,659 | 34,023 | 159,457 | 124,526 | 200,055 | 156,877 | 80,080 |
| MA | 4,330 | 8,421 | 12,521 | 11,243 | 3,340 | 9,889 | 7,069 | 16,061 | 8,146 | 3,948 | 6,716 | 13,923 | 6,781 | 4,442 | 3,614 | 9,671 | 29,860 | 8,934 |
| MG | 8,582 | 40,537 | 58,898 | 23,355 | 20,562 | 20,053 | 41,896 | 44,116 | 79,645 | 83,121 | 266,620 | 65,933 | 50,495 | 501,676 | 94,487 | 258,073 | 665,938 | 61,243 |
| MS | 6,430 | 12,104 | 18,777 | 6175 | 1,782 | 2,383 | 18,141 | 75,618 | 5,175 | 21,105 | 86,703 | 16,185 | 17,879 | 98,893 | 9,761 | 47,585 | 60,383 | 6,983 |
| MT | 350 | 4,586 | 14,645 | 14,194 | 4,502 | 11,716 | 15,768 | 21,033 | 11,615 | 63,473 | 45,586 | 10,107 | 43,537 | 45,192 | 12,033 | 32,136 | 31,084 | 12,131 |
| PA | 10,009 | 18,838 | 14,741 | 14,857 | 9,874 | 10,931 | 9,499 | 20,458 | 2,6247 | 12,099 | 21,716 | 30,023 | 27,169 | 17,441 | 9,690 | 17,029 | 19,435 | 13,201 |
| PB | 25 | 16,347 | 22,144 | 16,094 | 2,467 | 7,902 | 3,893 | 12,790 | 11,351 | 1,611 | 8,663 | 16,300 | 11,543 | 17,963 | 7,651 | 29,926 | 44,626 | 4,502 |
| PE | 29,968 | 16,862 | 116,328 | 26,249 | 6,379 | 12,887 | 18,832 | 35,705 | 41,850 | 8,390 | 59,435 | 38,376 | 63,908 | 17,747 | 19,927 | 172,648 | 119,648 | 17,375 |
| PI | 7,561 | 11,521 | 11,882 | 12,598 | 1,628 | 7,766 | 7,062 | 13,681 | 5,241 | 5,930 | 8,780 | 13,144 | 16,132 | 7,072 | 9,897 | 11,230 | 8,498 | 7,908 |
| PR | 2,388 | 3,875 | 16,281 | 22,122 | 3,429 | 4,815 | 5,393 | 51,485 | 18,404 | 10,783 | 67,296 | 66,972 | 17,768 | 112,250 | 56,491 | 97,887 | 136,076 | 26,721 |
| RJ | 3,760 | 66,757 | 266,668 | 7,923 | 2,005 | 2,307 | 31,224 | 65,713 | 256,385 | 13,127 | 42,295 | 190,777 | 252,707 | 254,572 | 15,898 | 93,101 | 114,795 | 16,584 |
| RN | 17,736 | 39,972 | 24,793 | 22,692 | 3,435 | 6,850 | 9,643 | 15,886 | 42,833 | 3,893 | 9,402 | 28,439 | 35,583 | 24,594 | 14,140 | 28,312 | 63,297 | 9,389 |
| RO | 3,648 | 1,982 | 3,199 | 5,272 | 5,526 | 9,019 | 6,469 | 5,657 | 10,212 | 24,753 | 28,200 | 5,679 | 5,215 | 14,148 | 5,232 | 5,880 | 15,608 | 6,293 |
| RR | 72,44 | 6,328 | 4,147 | 7,751 | 1,969 | 5,070 | 2,424 | 2,577 | 7,527 | 6,154 | 11,744 | 2,981 | 4,580 | 2,225 | 2,222 | 2,746 | 1,935 | 4,762 |
| RS | 63 | 145 | 1,285 | 258 | 133 | 161 | 180 | 1,410 | 842 | 274 | 4,935 | 1,659 | 610 | 2,385 | 799 | 4,124 | 8,334 | 1,595 |
| SC | 75 | 165 | 1,066 | 304 | 188 | 222 | 270 | 588 | 721 | 254 | 651 | 727 | 439 | 1,154 | 696 | 11,490 | 14,302 | 2,588 |
| SE | 8,660 | 4,642 | 7,329 | 7,551 | 1,009 | 1,630 | 2,330 | 2,213 | 35,607 | 3,671 | 2,003 | 7,132 | 11,174 | 2,405 | 4,271 | 14,729 | 7,461 | 1,343 |
| SP | 11,394 | 92,821 | 93,683 | 40,755 | 13,773 | 19,991 | 108,215 | 187,530 | 72,399 | 44,047 | 304,879 | 232,649 | 96,550 | 403,573 | 386,062 | 1,031,897 | 464,135 | 83,390 |
| TO | 2,958 | 7,955 | 5,191 | 5,370 | 3,701 | 6,821 | 9,554 | 22,023 | 21,632 | 10,200 | 17,375 | 22,575 | 25,875 | 20,912 | 10,520 | 19,084 | 19,769 | 14,801 |

| **Table 2 – Text S3.** Comparison of performance of concurrent models according to the 12-month forecasting horizon. | | | | | | | | | | | | | | |
| --- | --- | --- | --- | --- | --- | --- | --- | --- | --- | --- | --- | --- | --- | --- |
| **States** | **Models** | **MAPE** | **Scale (Naïve/ Model)** | **Theil's U** | **States** | **Models** | **MAPE** | **Scale (Naïve/ Model)** | **Theil's U** | **States** | **Models** | **MAPE** | **Scale (Naïve/ Model)** | **Theil's U** |
| *Acre* | *ELM* | 4.18 | 2.42 | 0.66 | *Ceará* | *NNETAR* | 4.1 | 3.22 | 0.88 | *Mato Grosso* | *ARIMA* | 3.72 | 3.04 | 0.96 |
|  | *BATS* | 4.72 | 2.14 | 0.77 |  | *ARIMA* | 4.59 | 2.87 | 0.88 |  | *ETS* | 4.00 | 2.83 | 0.99 |
|  | *MLP* | 5.51 | 1.84 | 0.9 |  | *TBATS* | 6.91 | 1.91 | 1.33 |  | *ELM* | 4.07 | 2.78 | 1.05 |
| *Amapá* | *ELM* | 8.59 | 1.44 | 1.12 | *Maranhão* | *ARIMA* | 2.96 | 5.02 | 0.41 | *Mato Grosso do Sul* | *ELM* | 5.21 | 1.06 | 1.30 |
|  | *Naïve* | 12.41 | 1.00 | 1.53 |  | *TBATS* | 4.27 | 3.48 | 0.51 |  | *Naïve* | 5.53 | 1.00 | 1.28 |
|  | *MLP* | 14.59 | 0.85 | 1.85 |  | *ETS* | 5.47 | 2.71 | 0.68 |  | *StructTS* | 6.75 | 0.82 | 1.59 |
| *Amazonas* | *STLM* | 3.65 | 1.99 | 1.38 | *Paraíba* | *TBATS* | 8.03 | 3.32 | 1.51 | *Espirito Santo* | *Naïve* | 9.73 | 1.00 | 2.47 |
|  | *ARIMA* | 4.43 | 1.64 | 1.47 |  | *ARIMA* | 8.23 | 3.24 | 1.45 |  | *MLP* | 10.82 | 0.90 | 2.59 |
|  | *ELM* | 4.88 | 1.49 | 1.81 |  | *NNETAR* | 9.19 | 2.90 | 1.88 |  | *ELM* | 11.3 | 0.86 | 2.45 |
| *Pará* | *STLM* | 4.43 | 2.23 | 0.97 | *Pernambuco* | *ELM* | 3.51 | 2.46 | 1.13 | *Minas Gerais* | *NNETAR* | 4.48 | 1.89 | 1.88 |
|  | *ETS* | 4.53 | 2.18 | 1.00 |  | *STLM* | 4.64 | 1.86 | 1.74 |  | *Naïve* | 8.48 | 1.00 | 2.12 |
|  | *TBATS* | 5.02 | 1.96 | 1.21 |  | *TBATS* | 5.54 | 1.56 | 1.88 |  | *STLM* | 8.11 | 1.05 | 2.52 |
| *Rondônia* | *TBATS* | 6.5 | 3.29 | 1.50 | *Piauí* | *ARIMA* | 5.66 | 3.60 | 0.80 | *Rio de Janeiro* | *ELM* | 5.62 | 1.39 | 1.86 |
|  | *ETS* | 6.89 | 3.10 | 1.63 |  | *STLM* | 5.51 | 3.69 | 0.67 |  | *Naïve* | 7.83 | 1.00 | 2.75 |
|  | *BATS* | 7.13 | 3.00 | 1.70 |  | *BATS* | 5.97 | 3.41 | 0.77 |  | *TBATS* | 7.52 | 1.04 | 3.01 |
| *Roraima* | *BATS* | 11.99 | 2.07 | 1.65 | *Rio Grande do Norte* | *ETS* | 5.42 | 2.38 | 1.48 | *São Paulo* | *Naïve* | 5.56 | 1.00 | 1.94 |
|  | *TBATS* | 14.26 | 1.74 | 2.06 |  | *BATS* | 6.24 | 2.07 | 1.81 |  | *NNETAR* | 7.69 | 0.72 | 2.40 |
|  | *ARIMA* | 15.43 | 1.60 | 2.14 |  | *STLM* | 6.67 | 1.94 | 1.83 |  | *StructTS* | 8.54 | 0.65 | 2.77 |
| *Tocantins* | *NNETAR* | 7.87 | 1.82 | 1.48 | *Sergipe* | *StructTS* | 18.38 | 1.07 | 2.26 | *Paraná* | *Naïve* | 7.77 | 1.00 | 2.17 |
|  | *ARIMA* | 10.38 | 1.38 | 1.55 |  | *Naïve* | 19.66 | 1.00 | 2.43 |  | *MLP* | 9.09 | 0.85 | 2.42 |
|  | *STLM* | 10.37 | 1.38 | 1.63 |  | *ELM* | 21.04 | 0.93 | 2.40 |  | *ARIMA* | 9.29 | 0.84 | 2.57 |
| *Alagoas* | *ELM* | 5.16 | 1.64 | 1.00 | *Distrito Federal* | *STLM* | 4.52 | 2.25 | 0.74 | *Rio Grande do Sul* | *NNETAR* | 14.8 | 1.88 | 2.25 |
|  | *STLM* | 7.86 | 1.08 | 1.43 |  | *TBATS* | 5.88 | 1.73 | 0.99 |  | *MLP* | 16.33 | 1.71 | 2.34 |
|  | *ETS* | 8.64 | 0.98 | 1.67 |  | *ETS* | 6.1 | 1.66 | 1.06 |  | *BATS* | 18.07 | 1.54 | 2.79 |
| *Bahia* | *Naïve* | 9.72 | 1.00 | 3.11 | *Goiás* | *STLM* | 4.01 | 2.16 | 1.06 | *Santa Catarina* | *TBATS* | 9.39 | 1.72 | 1.87 |
|  | *ELM* | 11.03 | 0.88 | 3.26 |  | *NNETAR* | 4.29 | 2.02 | 1.18 |  | *ARIMA* | 10.95 | 1.48 | 2.45 |
|  | *NNETAR* | 10.93 | 0.89 | 3.84 |  | *ELM* | 4.55 | 1.90 | 1.36 |  | *StructTS* | 14.85 | 1.09 | 3.35 |

| **Table 3 – Text S3.** Comparison of performance of concurrent models according to the 6-month forecasting horizon. | | | | | | | | | | | | | | |
| --- | --- | --- | --- | --- | --- | --- | --- | --- | --- | --- | --- | --- | --- | --- |
| **States** | **Models** | **MAPE** | **Scale (Naïve/ Model)** | **Theil's U** | **States** | **Models** | **MAPE** | **Scale (Naïve/ Model)** | **Theil's U** | **States** | **Models** | **MAPE** | **Scale (Naïve/ Model)** | **Theil's U** |
| *Acre* | *TBATS* | 2.27 | 4.15 | 0.31 | *Ceará* | *ARIMA* | 4.25 | 6.01 | 0.97 | *Mato Grosso* | *NNETAR* | 3.43 | 3.07 | 1.02 |
|  | *NNETAR* | 2.94 | 3.21 | 0.42 |  | *NNETAR* | 4.71 | 5.43 | 1.2 |  | *STLM* | 5.98 | 1.76 | 1.61 |
|  | *BATS* | 3.35 | 2.81 | 0.45 |  | *STLM* | 5.8 | 4.41 | 1.23 |  | *ARIMA* | 6.40 | 1.65 | 1.64 |
| *Amapá* | *ETS* | 4.68 | 3.54 | 0.51 | *Maranhão* | *STLM* | 1.9 | 11.37 | 0.37 | *Mato Grosso do Sul* | *ELM* | 4.64 | 1.08 | 0.81 |
|  | *TBATS* | 7.1 | 2.33 | 0.81 |  | *TBATS* | 3.15 | 6.86 | 0.66 |  | *Naïve* | 4.99 | 1.00 | 0.92 |
|  | *STLM* | 8.13 | 2.04 | 0.96 |  | *ARIMA* | 3.62 | 5.97 | 0.73 |  | *StructTS* | 6.35 | 0.79 | 1.38 |
| *Amazonas* | *Naïve* | 2.68 | 1.00 | 1.34 | *Paraíba* | *Naïve* | 4.81 | 1.00 | 1.06 | *Espirito Santo* | *ARIMA* | 5.72 | 1.54 | 2.43 |
|  | *STLM* | 5.76 | 0.47 | 2.95 |  | *ELM* | 9.26 | 0.52 | 2.33 |  | *StructTS* | 6.81 | 1.29 | 3.17 |
|  | *ARIMA* | 5.77 | 0.46 | 2.94 |  | *ARIMA* | 10.01 | 0.48 | 2.24 |  | *STLM* | 7.28 | 1.21 | 2.98 |
| *Pará* | *STLM* | 4.02 | 1.20 | 1.28 | *Pernambuco* | *ARIMA* | 3.23 | 3.69 | 1.40 | *Minas Gerais* | *ARIMA* | 4.00 | 1.56 | 2.79 |
|  | *ETS* | 4.66 | 1.04 | 1.64 |  | *BATS* | 3.38 | 3.53 | 1.32 |  | *ELM* | 4.35 | 1.43 | 2.49 |
|  | *Naïve* | 4.83 | 1.00 | 1.41 |  | *TBATS* | 3.89 | 3.07 | 1.60 |  | *Naïve* | 6.22 | 1.00 | 2.72 |
| *Rondônia* | *ELM* | 6.96 | 1.38 | 1.70 | *Piauí* | *TBATS* | 6.37 | 6.92 | 0.91 | *Rio de Janeiro* | *ARIMA* | 3.24 | 2.71 | 1.15 |
|  | *MLP* | 7.41 | 1.29 | 1.99 |  | *BATS* | 6.55 | 6.73 | 0.95 |  | *ETS* | 6.15 | 1.43 | 2.24 |
|  | *STLM* | 7.41 | 1.29 | 2.30 |  | *ARIMA* | 7.42 | 5.94 | 1.09 |  | *TBATS* | 6.18 | 1.42 | 2.24 |
| *Roraima* | *ARIMA* | 11.71 | 2.40 | 1.39 | *Rio Grande do Norte* | *StructTS* | 1.58 | 3.30 | 0.44 | *São Paulo* | *ELM* | 3.88 | 1.40 | 1.24 |
|  | *TBATS* | 15.25 | 1.85 | 1.71 |  | *TBATS* | 4.92 | 1.06 | 1.36 |  | *Naïve* | 5.42 | 1.00 | 1.40 |
|  | *BATS* | 15.64 | 1.80 | 1.75 |  | *Naïve* | 5.22 | 1.00 | 1.57 |  | *NNETAR* | 6.08 | 0.89 | 2.03 |
| *Tocantins* | *NNETAR* | 9.07 | 2.84 | 1.65 | *Sergipe* | *ETS* | 6.81 | 2.79 | 0.75 | *Paraná* | *ARIMA* | 3.46 | 2.42 | 0.91 |
|  | *StructTS* | 9.69 | 2.66 | 2.34 |  | *STLM* | 8.29 | 2.30 | 0.79 |  | *BATS* | 4.68 | 1.79 | 1.33 |
|  | *ARIMA* | 10.18 | 2.53 | 2.03 |  | *MLP* | 14.14 | 1.35 | 1.57 |  | *ELM* | 5.77 | 1.45 | 1.60 |
| *Alagoas* | *STLM* | 5.21 | 4.36 | 1.28 | *Distrito Federal* | *STLM* | 4.99 | 6.04 | 1.81 | *Rio Grande do Sul* | *ELM* | 3.94 | 1.86 | 1.06 |
|  | *ETS* | 6.15 | 3.69 | 1.42 |  | *MLP* | 9.83 | 3.07 | 3.82 |  | *MLP* | 4.86 | 1.51 | 1.44 |
|  | *TBATS* | 7.12 | 3.19 | 1.68 |  | *NNETAR* | 9.74 | 3.10 | 3.04 |  | *ARIMA* | 6.39 | 1.15 | 1.80 |
| *Bahia* | *StructTS* | 2.18 | 5.21 | 0.59 | *Goiás* | *NNETAR* | 3.98 | 4.58 | 1.49 | *Santa Catarina* | *StructTS* | 5.25 | 1.05 | 2.13 |
|  | *TBATS* | 4.59 | 2.47 | 1.96 |  | *StructTS* | 5.94 | 3.07 | 1.95 |  | *Naïve* | 5.51 | 1.00 | 2.16 |
|  | *ETS* | 4.59 | 2.47 | 1.31 |  | *STLM* | 6.99 | 2.61 | 2.63 |  | *BATS* | 6.26 | 0.88 | 2.51 |

| **Table 4 – Text S3.** Comparison of performance of concurrent models according to the 3-month forecasting horizon. | | | | | | | | | | | | | | |
| --- | --- | --- | --- | --- | --- | --- | --- | --- | --- | --- | --- | --- | --- | --- |
| **States** | **Models** | **MAPE^1^** | **Scale (Naïve/ Model)** | **Theil's U** | **States** | **Models** | **MAPE** | **Scale (Naïve/ Model)** | **Theil's U** | **States** | **Models** | **MAPE** | **Scale (Naïve/ Model)** | **Theil's U** |
| *Acre* | *BATS* | 2.93 | 6.94 | 0.29 | *Ceará* | *STLM* | 6.37 | 1.66 | 1.51 | *Mato Grosso* | *ARIMA* | 3.98 | 1.90 | 0.85 |
|  | *TBATS* | 4.15 | 4.90 | 0.43 |  | *ELM* | 7.36 | 1.43 | 1.64 |  | *ELM* | 4.91 | 1.54 | 1.23 |
|  | *ETS* | 4.27 | 4.76 | 0.52 |  | *ETS* | 8.15 | 1.30 | 1.81 |  | *STLM* | 5.17 | 1.47 | 1.27 |
| *Amapá* | *ETS* | 5.19 | 3.05 | 0.17 | *Maranhão* | *ETS* | 0.89 | 5.54 | 0.25 | *Mato Grosso do Sul* | *ELM* | 0.53 | 1.04 | 0.94 |
|  | *StructTS* | 7.21 | 2.19 | 0.45 |  | *BATS* | 1.43 | 3.45 | 0.55 |  | *Naïve* | 0.55 | 1.00 | 0.71 |
|  | *STLM* | 10.31 | 1.53 | 0.72 |  | *ARIMA* | 1.48 | 3.33 | 0.6 |  | *StructTS* | 1.26 | 0.44 | 1.73 |
| *Amazonas* | *ELM* | 0.85 | 3.98 | 0.30 | *Paraíba* | *Naïve* | 2.47 | 1.00 | 0.75 | *Espirito Santo* | *MLP* | 0.81 | 2.06 | 0.52 |
|  | *MLP* | 1.56 | 2.17 | 0.47 |  | *ETS* | 2.63 | 0.94 | 0.61 |  | *ELM* | 1.29 | 1.29 | 0.54 |
|  | *Naïve* | 3.38 | 1.00 | 1.45 |  | *ELM* | 6.18 | 0.40 | 1.87 |  | *Naïve* | 1.67 | 1.00 | 1.04 |
| *Pará* | *Naïve* | 2.41 | 1.00 | 0.81 | *Pernambuco* | *ELM* | 0.51 | 11.94 | 0.18 | *Minas Gerais* | *StructTS* | 1.75 | 2.36 | 1.63 |
|  | *MLP* | 2.62 | 0.92 | 0.75 |  | *StructTS* | 1.64 | 3.71 | 0.72 |  | *ELM* | 1.76 | 2.35 | 2.04 |
|  | *STLM* | 5.23 | 0.46 | 1.91 |  | *STLM* | 2.00 | 3.05 | 0.62 |  | *STLM* | 3.47 | 1.19 | 3.39 |
| *Rondônia* | *ELM* | 2.86 | 2.83 | 0.53 | *Piauí* | *StructTS* | 4.59 | 5.23 | 0.73 | *Rio de Janeiro* | *STLM* | 3.26 | 1.41 | 1.53 |
|  | *StructTS* | 7.09 | 1.14 | 2.18 |  | *MLP* | 6.02 | 3.99 | 1.22 |  | *BATS* | 3.32 | 1.39 | 2.37 |
|  | *Naïve* | 8.1 | 1.00 | 2.42 |  | *ETS* | 8.34 | 2.88 | 1.73 |  | *ARIMA* | 3.46 | 1.33 | 3.25 |
| *Roraima* | *NNETAR* | 5.59 | 1.03 | 0.43 | *Rio Grande do Norte* | *StructTS* | 0.82 | 3.89 | 0.14 | *São Paulo* | *MLP* | 1.85 | 1.46 | 1.21 |
|  | *MLP* | 5.55 | 1.04 | 0.37 |  | *ELM* | 2.35 | 1.36 | 0.66 |  | *BATS* | 1.93 | 1.40 | 0.97 |
|  | *BATS* | 6.07 | 0.95 | 0.81 |  | *Naïve* | 3.19 | 1.00 | 1.19 |  | *ELM* | 2.03 | 1.33 | 1.56 |
| *Tocantins* | *Naïve* | 4.37 | 1.00 | 0.75 | *Sergipe* | *MLP* | 7.73 | 1.80 | 0.58 | *Paraná* | *ELM* | 0.57 | 4.49 | 0.74 |
|  | *ELM* | 4.74 | 0.92 | 0.51 |  | *ETS* | 11.17 | 1.25 | 0.87 |  | *MLP* | 0.62 | 4.13 | 0.75 |
|  | *MLP* | 6.32 | 0.69 | 1.1 |  | *STLM* | 11.08 | 1.26 | 0.82 |  | *TBATS* | 1.72 | 1.49 | 2.81 |
| *Alagoas* | *ARIMA* | 1.72 | 2.01 | 0.18 | *Distrito Federal* | Naïve | 1.67 | 1.00 | 1.09 | *Rio Grande do Sul* | *BATS* | 4.8 | 1.57 | 2.49 |
|  | *ETS* | 3.22 | 1.07 | 0.91 |  | ELM | 2.10 | 0.80 | 1.31 |  | *TBATS* | 6.05 | 1.25 | 3.25 |
|  | *ELM* | 3.24 | 1.07 | 0.85 |  | STLM | 4.10 | 0.41 | 2.7 |  | *ELM* | 6.65 | 1.14 | 3.36 |
| *Bahia* | *ELM* | 1.27 | 2.40 | 0.25 | *Goiás* | MLP | 0.37 | 7.22 | 0.44 | *Santa Catarina* | *ARIMA* | 0.84 | 4.48 | 0.46 |
|  | *MLP* | 1.77 | 1.72 | 0.64 |  | ELM | 2.58 | 1.03 | 3.73 |  | *STLM* | 2.66 | 1.41 | 1.57 |
|  | *Naïve* | 3.05 | 1.00 | 0.97 |  | Naïve | 2.67 | 1.00 | 2.17 |  | *ELM* | 3.28 | 1.15 | 1.72 |
